# Burnout of Healthcare Workers amid the COVID-19 Pandemic: A Japanese Cross-Sectional Survey

**DOI:** 10.3390/ijerph18052434

**Published:** 2021-03-02

**Authors:** Yoshito Nishimura, Tomoko Miyoshi, Hideharu Hagiya, Yoshinori Kosaki, Fumio Otsuka

**Affiliations:** 1 Department of General Medicine, Graduate School of Medicine, Dentistry and Pharmaceutical Sciences, Okayama University, Okayama 7008558, Japan; tmiyoshi@md.okayama-u.ac.jp (T.M.); hagiya@okayama-u.ac.jp (H.H.); fumiotsu@md.okayama-u.ac.jp (F.O.); 2 Department of Medicine, John A. Burns School of Medicine, University of Hawai’i, Honolulu, HI 96813, USA; 3 Center for Education in Medicine and Health Sciences, Graduate School of Medicine, Dentistry and Pharmaceutical Sciences, Okayama University, Okayama 7008558, Japan; pj4w35pr@okayama-u.ac.jp

**Keywords:** coronavirus disease 2019 (COVID-19), pandemic, burnout, prevention, healthcare delivery system, intention to leave

## Abstract

The coronavirus disease 2019 (COVID-19) global pandemic has drastically changed how we live and work. Amid the prolonged pandemic, burnout of the frontline healthcare professionals has become a significant concern. We conducted a cross-sectional survey study to provide data about the relationship between the COVID-19 pandemic and the prevalence of burnout in healthcare professionals in Japan. Healthcare workers in a single Japanese national university hospital participated in the survey, including basic demographics, whether a participant engaged in care of COVID-19 patients in the past 2 weeks and the Maslach Burnout Inventory. Of those, 25.4% fully answered the survey; 33.3% were doctors and 63.6% were nurses, and 36.3% engaged in care of COVID-19 patients in the past 2 weeks. Compared to those belonging to General Medicine, those in Emergency Intensive Care Unit were at higher risk of burnout (odds ratio (OR), 6.7; 95% CI, 1.1–42.1; *p* = 0.031). Of those who engaged in care of COVID-19 patients, 50% reported burnout while 6.1% did not (OR 8.5, 95% CI; 1.3–54.1; *p* = 0.014). The burnout of healthcare workers is a significant concern amid the pandemic, which needs to be addressed for sustainable healthcare delivery.

## 1. Introduction

The coronavirus disease 2019 (COVID-19) global pandemic has drastically changed how we live and work. According to the World Health Organization (WHO), more than 88 million cases and 1.9 million deaths have been reported worldwide as of 12 January 2021 [1]. Japan, which successfully controlled the pandemic at first [2], has experienced its third wave of the big surge in the number of COVID-19 patients since November 2020. As of 12 January 2021, 290,736 cumulative cases have been reported [3]. Amid the prolonged struggles against COVID-19, burnout, a chronic psychological condition with a loss of enthusiasm and personal accomplishment, feelings of physical and mental exhaustion, and depersonalization [4], of the frontline healthcare professionals has become a significant concern.

By its nature, burnout is prevalent among healthcare workers (HCWs) [5]. While there has been no nationwide data about the prevalence of burnout in Japanese HCWs, we previously reported that approximately 20–30% of resident physicians in Japan, who are considered to be more vulnerable to stress, were experiencing burnout before the COVID-19 pandemic [6]. Now, because of the pandemic, HCWs have been exposed to an unprecedented level of stress since March 2020, when the pandemic spread all over the world. Several studies have been conducted to look into the prevalence of and factors related to burnout [7,8,9,10,11,12,13,14,15]. However, there is noc uniform pattern about the factors related to burnout of HCWs. A study by Amanullah et al. reported that exposure to COVID-19 did not correlate with increased burnout [7]. In contrast, other cross-sectional studies noted that frequent contact with COVID-19 was the most important factor associated with psychological burden and burnout [13,14,15].

In Japan, Matsuo et al. conducted the first cross-sectional study to investigate the prevalence of burnout among HCWs in Japan in April 2020, which reported an overall prevalence of burnout of 31.4% [12]. The authors also noted that being non-physicians was one of the significant factors related to burnout. However, at the time of this study, when the country experienced its first COVID-19 surge, it had only 2178 cumulative COVID-19 cases as of 1 April 2020 [16]. Now, Japan is experiencing the third surge of COVID-19 cases, characterized by exponential growth in the number of cases and deaths related to COVID-19(3). Thus, the prevalence of burnout in HCWs could be totally different at this time. Moreover, the Japanese healthcare delivery system, which is famous for universal health coverage (UHC) [17,18], is now in danger of collapse, given the overwhelming number of COVID-19 hospitalizations.

To clarify the up-to-date prevalence of burnout in Japanese HCWs and mitigate, which may lead to further collapse of its sustainable healthcare system, we conducted a cross-sectional survey of HCWs working in a Japanese national university hospital.

## 2. Materials and Methods

### 2.1. Study Design, Setting, and Participants

We conducted a cross-sectional study that employed an anonymous, self-administered voluntary paper, or web-based survey. The participants comprised of physicians, nurses, and clinical engineers working in Okayama University Hospital (OUH; a Japanese national university hospital). We implemented a power analysis for two proportions to estimate a sample size needed to detect the significant differences between the prevalence of burnout among those engaged in the COVID-19 patient care in the past 2 weeks and those who did not. Based on previous studies [6,14,15], we anticipated the prevalence of burnout in those who engaged in COVID-19 care in the period as 70% and those who did not as 20%. With the level of significance of 5% and power of the test of 80%, the sample size needed was estimated as *n* = 15 in each group (*n* = 30 as a whole). We used a homogenous purposive sampling to survey the 130 healthcare professionals who belonged to the OUH on 1 November 2020. The purposive sampling criteria included those who belonged to the Department of General Medicine, Emergency Department/Intensive Care Unit (EICU), Center for Graduate Medical Education, who could potentially take care of COVID-19 patients of patients under investigation (PUI) of CIVUD-19 during the study period. Their completion of the survey implied the participants’ consent. The survey was developed through consultation with a medical education expert panel at OUH and piloting. We provided survey instructions and instruments in Japanese. All participants were invited to complete the survey within two weeks (13–30 November 2020, in Japan Standard Time). No financial incentives were provided for their participation in the survey. In the survey, we included entries on demographics (gender, job category, affiliation, years of experience, size of household) as well as COVID-19 related items (e.g., “Have you engaged in care of patients with COVID-19 or PUI of COVID-19 in the past 2 weeks?”). To protect participants’ anonymity as much as possible, the respondents were not prompted to enter their age.

### 2.2. Measurements

#### The Maslach Burnout Index (MBI)

Burnout was measured using the Japanese translation of the Maslach Burnout Inventory–Human Services Survey (MBI-HSS). This instrument was validated for measuring burnout by Higashiguchi et al. [19]. It consists of 22 items covering three domains: emotional exhaustion (EE), depersonalization (DP), and personal accomplishment (PA). Each item has a 7-point Likert scale from “never” or 0 to “daily” or 6. While there are no definite cut-off points for MBI subscales, based on a previous study that investigated the most commonly used raw score cut-off, we defined a 27 or higher EE score and a 10 or higher DP score (the most common cut-off) as burnout in healthcare professionals [20]. Since Maslach et al. noted that PA is an independent subscale that does not correlate with EE and DP subscales, we did not define low PA scores (33 or lower; the most commonly used cut-off) as burnout in the present study [21].

### 2.3. Statistical Analysis

We analyzed the data using JMP version 15.1.0 (SAS Institute Inc., Cary, NC, USA). We used the Mann–Whitney U test to examine differences in the time participants’ MBI scores based on non-normal distribution. For associations between categorical variables, we employed Fisher’s exact test. The threshold for significance was defined as *p* < 0.05.

## 3. Results

The response rates to the survey were 33 out of 130 (25.4%) OUH healthcare professionals. Participants’ demographic characteristics are summarized in Table 1. Of note, the mean years of experience of the participants were 11.5 (95% CI: 8.6–14.4). 11 (33.3%) were physicians, and 21 (63.6%) were nurses. 12 (36.4%) of the respondents answered that they had engaged in the direct care of COVID-19 patients or PUI in the past 2 weeks.

Table 2 and Table 3 show the respondents’ answers to the MBI and the prevalence of burnout, respectively. While the scores of EE and DP were higher in those who engaged in care of COVID-19 patients or PUI, no statistically significant differences were noted in these scores compared to those who did not engage in the care of COVID-19 patients or PUI in the past 2 weeks. The PA scores (higher score suggests less feeling of better self-accomplishment) tended to be higher in those who engaged in COVID-19 patients care than those who did not, but the difference was not statistically significant. Among those who did engage in the care of COVID-19 care in the last 2 weeks, 6 (50.0%) of them were experiencing burnout, while burnout was noted in 2 (9.5%) of those who did not. The difference was statistically significant based on Fisher’s exact test (*p* = 0.016). Compared to those belonging to General Medicine, those in EICU were at higher risk of burnout (odds ratio (OR), 6.7; 95% CI, 1.1–42.1; *p* = 0.031). Also, those who engaged in the COVID-19 care had significantly higher odds of experiencing burnout than those who did not (OR, 8.5; 95% CI, 1.3–54.1; *p* = 0.014). Other variables, including gender, years of experience, affiliation, job category, were not significant predictors of burnout. Of note, 3/11 (27.3%) of physicians and 5/21 (23.8%) of nurses were experiencing burnout (*p* = 0.828).

## 4. Discussion

In this study, we found that 50% of the front line HCWs who engaged in direct care for COVID-19 patients or PUI experienced burnout, which was a significantly higher prevalence than those who did not participate in the care of COVID-19 patients. Although Japan has had relatively fewer COVID-19 cases than most of the other developed countries, the study results underscore that the COVID-19 pandemic has rendered profound stress on our society and HCWs. Also, according to the most recent statistics of the Japanese Ministry of Health, Labour and Welfare, 21.9% (among 327,210 physicians) and 92.8% (among 1,218,606 nurses) were female, suggesting that 77.3% of physicians and nurses were female in Japan [22,23]. Our study included 72.7% of female HCWs with a predominance in nurses. Thus, the occupational and gender distributions were similar to that of the Japanese HCWs as a whole, which raises the study’s generalizability despite the small sample size.

Compared to the previous cross-sectional study that looked into the prevalence of burnout in Japan in April 2020 [12], our data shows that engagement in the care of COVID-19 patients or PUI is a significant factor correlated with burnout, with OR 8.5 compared to those who did not see these patients. Also, those in the EICU had higher odds of burnout. While a previous study noted that those working in Emergency Medicine could have a high prevalence of burnout at the baseline due to the demanding nature of their work and patient care [24], direct care and supervision of COVID-19 patients involve greater substantial physical and psychological burdens on HCWs due to a need for thorough observation of infection prevention and control practices and unfamiliarity to manage severe COVID-19 cases. Thus, the results were as we expected.

Now, what is the most substantial problem of burnout? Burnout and occupational stress have been known to affect the intention to leave the profession through decreased job satisfaction, the sense of being overwhelmed, and potential depression and anxiety [25,26]. Although Japan has been a world leader to promote UHC in global health [17,18,27], its healthcare delivery system is now at the stake of collapse due to the overwhelming number of COVID-19 hospitalization and related deaths. Before the pandemic, Japanese people believed that healthcare was equally available to all citizens regardless of their incomes, affiliations, or whereabouts [28]. Now, the Japanese healthcare system might not be able to measure up to these expectations. The potential leave of HCWs due to burnout exacerbated by COVID-19 might further accelerate the healthcare collapse and fragmentation of health systems, leading to inequities and disruption of UHC [29].

While some mitigating interventions including mindfulness, counseling those at risk of burnout, and reducing workload have been proposed as measures to address the widespread burnout of HCWs [9,10,13,15], given the unprecedented surge in the number of COVID-19 cases, individual efforts might not be sufficient to overcome the issue. Recently, psychological resilience has been highlighted as a protective factor against burnout [30,31,32,33]. Psychological resilience is defined as the ability of individuals to adapt and respond to difficulties. A recent study from Portugal noted that resilience could be a partial mediator between depression and burnout, with high resilience being protective against burnout [32]. For now, we call for organizational or policy-level approaches to protect HCWs from burnout. Although there are no simple solutions to burnout preventions, securing shift work to prevent overwork and providing mental health support would need to be addressed at a high-level policy level to make them more practical and effective. Also, given the importance of nurturing psychological resilience, it is crucial to provide psychological training and interventions to HCWs skills to address emotional challenges. When it comes to providing prevention intervention on resilience, it is also essential to focus on flexibility in emotional response, which was noted as a significant factor of emotional exhaustion [34,35]. Future studies are warranted to investigate the prevalence of burnout with a focus on the psychological resilience of Japanese HCWs using validated instruments.

Several limitations to this study need to be noted. First, due to the single-center cross-sectional survey design with the small sample size, we may not be able to conclude the causal relationship between burnout and engagement in the care of COVID-19 patients. Also, the results might have been biased by organizational climate, which was suggested to be a predictor of burnout [36]. Second, cross-sectional research has a limitation in terms of addressing changes over time. A longitudinal study design needs to be pursued to clarify the pandemic’s long-term effect on the burnout of HCWs. Third, the survey included 14 EICU physicians and 58 EICU nurses, while only 8 responded. Since those who worked in the EICU had significantly higher odds of burnout, the true prevalence of burnout among the study population might have been higher. Lastly, due to the nature of the survey topic, which focused more on preventive and occupational medicine, HCWs who were interested in these topics might have been more likely to respond, which could lead to self-selection bias. Despite these limitations, our study may be referred to as a first preliminary study to look into the burnout prevalence among the front line HCWs during the surge of COVID-19 cases in Japan.

## 5. Conclusions

Through the study, we provided up-to-date data regarding the impacts of the COVID-19 pandemic on HCWs focusing on burnout. Despite the emergence of novel vaccines, there is considerable uncertainty about how long the pandemic will last. While Japan has had a relatively small number of COVID-19 cases compared to other countries, we have experienced considerable numbers of clusters and outbreaks all over the countries that have rendered significant stress on HCWs. Burnout of HCWs, which is related to intention to leave the professions, would eventually lead to a chaotic consequence of the potential collapse of its sustainable healthcare system. Although there are no straightforward solutions to address burnout of HCWs, securing shift work to prevent overwork and providing mental health support at a policy level may be essential to overcome the challenge and to provide contingency plans to those in danger of burnout. As a country where UHC is an essential pillar of its policy, the Japanese government needs to show its leadership to protect its sustainable healthcare delivery system. One of the most important things to address the issue would be burnout prevention of HCWs. We call for high-level leadership to provide comprehensive support for HCWs amid the pandemic.

## Figures and Tables

**Table 1:** Demographic characteristics of the study participants.

Characteristic	Value	SD	95% CI
Years in experience			
Mean	11.5	8.2	8.6–14.4
Gender, no. (%)			
Female	24 (72.7)		
Male	9 (27.3)		
Affiliation, no. (%)			
Emergency department/Intensive care unit	8 (24.2)		
General Medicine	24 (72.7)		
Radiology	1 (0.3)		
Job category, no. (%)			
Physician	11 (33.3)		
Nurse	21 (63.6)		
Clinical engineer	1 (0.3)		
Marital status, no. (%)			
Single	12 (36.4)		
Married	21 (63.6)		
Size of household			
Mean	2.2	1.1	1.8–2.6
Engaged in care of COVID-19 patients or COVID-19 PUI in the past 2 weeks, no. (%)			
No	21 (63.6)		
Yes	12 (36.4) ^a^		
Total number of participants	33		

Abbreviations: CI, confidence interval; SD, standard deviation; PUI, patients under investigation. ^a^ Among those who answered “Yes” to the question, 6 of them were from the Department of General Medicine, and the rest of the 6 were from the Emergency department/Intensive care unit.

**Table 2:** Results of the Maslach Burnout Index.

Measure	Those Engaged in COVID-19 Care in the Last 2 Weeks ^a^ (*n* = 12)		Those Not Engaged in COVID-19 Care in the Last 2 Weeks ^a^ (*n* = 21)		*p* Value ^b^
	Mean	95% CI	Mean	95% CI	
Emotional exhaustion (EE)	24.8	18.8–30.7	18.0	13.5–22.4	0.116
Depersonalization (DP)	6.6	3.9–9.2	3.8	1.8–5.8	0.207
Personal accomplishment (PA)	27.4	22.0–32.8	21.8	17.7–25.9	0.088
Measure	No. (%)		No. (%)		
Burnout					0.016
Yes	6 (50.0)		2 (9.5)		
EE ≥ 27 DP ≥ 10	3/6 (50.0) 4/6 (66.7)		2/2 (100) 1/2 (50.0)		
No	6 (50.0)		19 (90.5)		

Abbreviations: CI, confidence interval. ^a^ The base date of the “last 2 weeks” is the day when the participants answered the survey. The survey period was from 13–30 November 2020. ^b^ *p* value is calculated with Mann-Whitney U test or Fisher’s exact test.

**Table 3:** Prevalence of burnout depending on variables.

Variable	OR (95% CI)	*p* Value
Gender		
Male	1 (Reference)	NA
Female	0.53 (0.10–2.8)	0.651
Job category		
Physician	1 (Reference)	NA
Nurse	1.2 (0.23–6.3)	0.830
Affiliation		
General Medicine	1 (Reference)	NA
EICU	6.7 (1.1–42.1)	0.031
Marital status		
Single	1 (Reference)	NA
Married	0.93 (0.18–4.9)	0.935
Engagement in COVID-19 care in the last 2 weeks		
No	1 (Reference)	NA
Yes	8.5 (1.3–54.1)	0.014

Abbreviations: CI, confidence interval; NA, not applicable; OR, odds ratio; EICU, Emergency Intensive Care Unit. The survey period was from 13–30 November 2020.

## Data Availability

The datasets generated and analyzed during the current study are available from the corresponding authors on reasonable request.
